# Accuracy assessment of Global Human Settlement Layer (GHSL) built-up products over China

**DOI:** 10.1371/journal.pone.0233164

**Published:** 2020-05-29

**Authors:** Feng Liu, Shuai Wang, Yi Xu, Qing Ying, Fukun Yang, Yuchu Qin

**Affiliations:** 1 State Key Laboratory of Remote Sensing Science, Aerospace Information Research Institute, Chinese Academy of Sciences, Beijing, China; 2 School of Electronic Electrical and Communication Engineering, University of Chinese Academy of Sciences, Beijing, China; 3 Department of Geographical Sciences, University of Maryland, College Park, Maryland, United States of America; Xiamen University, CHINA

## Abstract

Building a density map over large areas could provide essential information of land development intensity and settlement condition. It is crucial for supporting studies and planning of human settlement environment. The Global Human Settlement Layer (GHSL) is a comprehensive data set of mapping human settlement at a global scale, which was produced by the Joint Research Centre (JRC), European Commission. The built-up density is an important layer of GHSL data set. Currently, the validation of the GHSL built-up area products was preliminarily conducted over the United States and European countries. However, as a typical East Asian region, China is quite different from the United States, Europe, and other regions in terms of building forms and urban layouts. Therefore, it is necessary to perform an accuracy assessment of GHSL data set in Asian countries like China. With individual building footprint data of 20 typical cities in China, this paper presents our effort to validate the GHSL built-up area products. The aggregation mean and neighborhood search based algorithms are adopted for matching building footprint data and the GHSL products, through the regression analysis at per-pixel level, the building density map in raster format are generated as validation data. The accuracy index of GHSL built-up area was calculated for the study areas, and the validation methods were explored for GHSL built-up products at large scale. The results show that the built-up layer aggregated by the building footprint have the highest correlation with the coarse resolution GHSL built-up products, but GHSL tends to underestimate the building density of low-density areas and overestimate the areas with high density. This study suggests that GHSL built-up area products in 20 representative Chinese cities of China could provide quantitative information about built-up areas, but the product accuracy still need to be improved in the regions with heterogeneous formations of human settlements like China. There is a big picture of mapping high accuracy built-up density of China with the training data set acquired by the study.

## Introduction

Land cover is an important factor of environmental studies of the earth surfaces [1–3], since the land cover/land use change, environmental pollution, land degradation and loss of biodiversity have become increasingly serious. Thus, timely and reliable global land cover data has become an important data set for ecosystem assessment, environmental modeling, etc. [4]. Urbanization is one of the most significant factors that human influence the land cover of earth surfaces. Presently, more than 50% of the world’s population live in urban areas, compared with which is only 30% in 1950, and it is projected that the urban population will account for 66% of the world population by 2050 [5]. Although cities cover only a relatively small portion of the earth surfaces, studies in urban areas play a crucial role in human housing demand, climate change and response, disaster risk prevention, urban development and other sustainable development goals [6].

Currently, it is well addressed that remote sensing technology is a promising solution for large-area observation [7–9]. Satellite remote sensing has become an important means of obtaining information on land surfaces [10–12]. As the high-resolution remote sensing satellite data can provide detailed urban surfaces, Xu investigated the land cover information extraction using IKONOS panchromatic data with 1m resolution [13]. In [14], the principal component analysis is employed to fuse the texture and structure features derived from Landsat-7 ETM+ panchromatic data to extract the building information. Currently, the Global Urban Footprint (GUF) dataset is produced for urban mapping, it is based on the satellite SAR imagery acquired by the German satellites TerraSAR-X and TanDEM-X. With a fully automated processing system, global coverage of more than 180,000 very high-resolution SAR images with 3m ground resolution, mainly acquired between 2010 and 2013, were processed, the scattering amplitude is combined with the derived texture information to depict the human settlement. In addition, auxiliary data such as digital elevation models were fused with the SAR images to improve the classification accuracy [15]. The Global Human Built-up And Settlement Extent (HBASE) Dataset is a global scale product derived from the Global Land Survey (GLS) Landsat dataset for the year of 2010 [16], the product is only for the mapping and monitoring of urbanization. The Global Human Settlement Layer (GHSL) produced by the Joint Research Centre (JRC) provides much more detailed information on the growth of buildings and populations over the past 40 years (1975–2015) [17]. The products contain comprehensive data layer for urbanization assessment, land cover change, urban planning and management [6], species changes studies [18], but the accuracy of the product needs further validation. With urban building density information, many applications would be able to carry out, e.g. the economic development of the city and the expansion of urban space. Combining with urban lighting data and urban traffic data, the future urban development can also be predicted, which is of great significance to integrate urban management and improvement of the urban environment [12, 19–21]. However, there is no well validated global-scale human settlement mapping products for ecological environment studies [22]. Therefore, the development of large-scale building density remote sensing products, the formation of large-scale and long time series of remote sensing mapping products has become an urgent need for both research and applications.

The GHSL built-up area products are promising data collection set for characterizing the built-up area at large scale. However, GHSL is an experimental product, it is currently validated only in the United States and European countries [10]. Due to differences in population density and building structure between Asia and Europe, the validation results of GHSL built-up area products in the United States and Europe cannot assure the reliability of the accuracy in Asia. Therefore, this paper focuses on the validation and analysis of the accuracy of GHSL built-up area products in 20 representative Chinese cities in China. It is expected to provide reference accuracy information for applications of GHSL data in China. Based on the maps of building footprint derived from open geospatial web service in China, i.e., Baidu Map. The accuracy of the GHSL built-up area products at 250m resolution and 1000m resolution in 20 typical Chinese cities across different provinces were quantitatively assessed. The accuracy of GHSL built-up products in China is evaluated by aggregating the building footprint into building density products with the same resolution as GHSL built-up products. The results demonstrate that there is a certain misestimation in GHSL products over 20 representative Chinese cities. The results are expected to provide quantitative accuracy information of the GHSL built-up products application in China.

## Study area and data sets

### Study area

**Fig 1:**
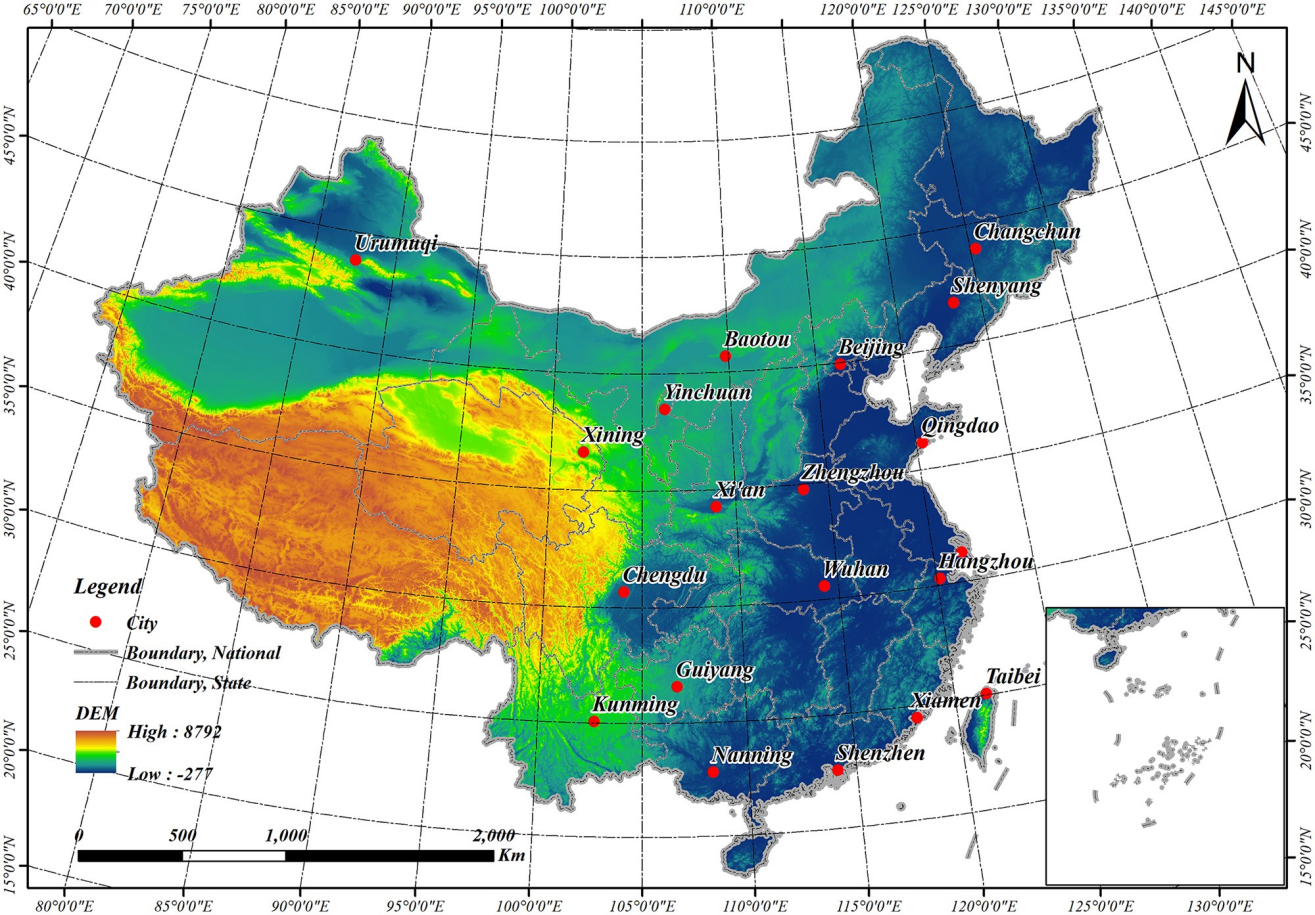
Distribution map of the study area.

To better represent the different urban patterns and building forms, this paper selected 20 typical cities as study area located in different administrative regions of China with varieties of economic developments, population densities and physical environments (Fig 1, Table 1). The building density patterns from GHSL in 20 cities of China were validated in this paper.

**Table 1:** GDP and population of each city.

FID	Longitude	Latitude	City name	GDP (Billion)	Population (Million)
1	87.62	43.82	Urumuqi	245.898	2.6787
2	116.4	39.9	Beijing	2566.91	13.629
3	108.93	34.27	Xi’an	625.718	8.2493
4	104.07	30.67	Chengdu	1217.02	13.989
5	102.72	25.05	Kunming	430.008	5.5979
6	121.47	31.23	Shanghai	2817.87	14.5
7	123.43	41.8	Shenyang	546.001	7.344
8	121.5	25.03	Taibei	541.265	2.75
9	114.3	30.6	Wuhan	1191.26	8.3385
10	114.05	22.55	Shenzhen	1949.26	3.8452
11	109.83	40.65	Baotou	386.76	2.6504
12	125.32	43.9	Changchun	591.794	7.5343
13	120.38	36.07	Qingdao	1001.13	7.9135
14	118.08	24.48	Xiamen	378.427	2.2055
15	120.15	30.28	Hangzhou	1131.37	7.36
16	113.62	34.75	Zhengzhou	802.531	8.2706
17	108.37	22.82	Nanning	370.339	7.5175
18	106.63	26.65	Guiyang	315.77	4.0135
19	101.78	36.62	Xining	124.817	2.0328
20	106.28	38.47	Yinchuan	161.771	1.8404

### GHSL built-up areas products

**Fig 2:**
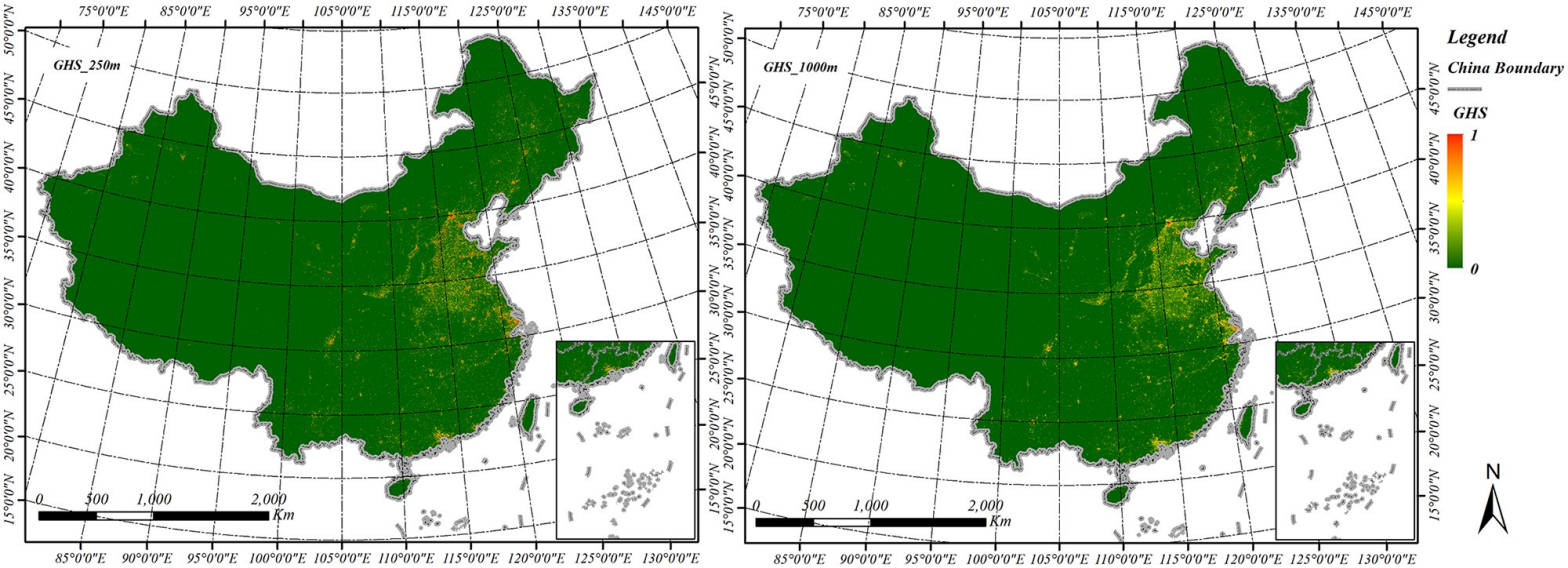
GHSL built-up map products of China. (a) The GHSL built-up areas with 250m resolution, (b) The GHSL built-up areas with 1000m resolution.

GHSL is a global scale human settlement map product extracted from Landsat images, it is developed with a classification method based on symbolic machine learning [23, 24]. To utilize long term remote sensing data record, GHSL adopts images acquired by the Multi-Spectral Scanner (MSS) and Thematic Mapper (TM), Enhanced Thematic Mapper (ETM+), Operational Land Imager (OIL) and Digital Elevation Model (DEM) for characterizing human settlement. The time series consisted mostly of four epochs, 1975, 1990, 2000, and 2014. The built-up area products in each epoch are provided with the resolution of 38m, 250m and 1000m. This paper selects the latest GHSL product data—built-up area products in China with the resolution of 250m and 1000m in 2014 for the validation. Fig 2 illustrates the GHSL built-up area products in China.

### Building footprint data

**Fig 3:**
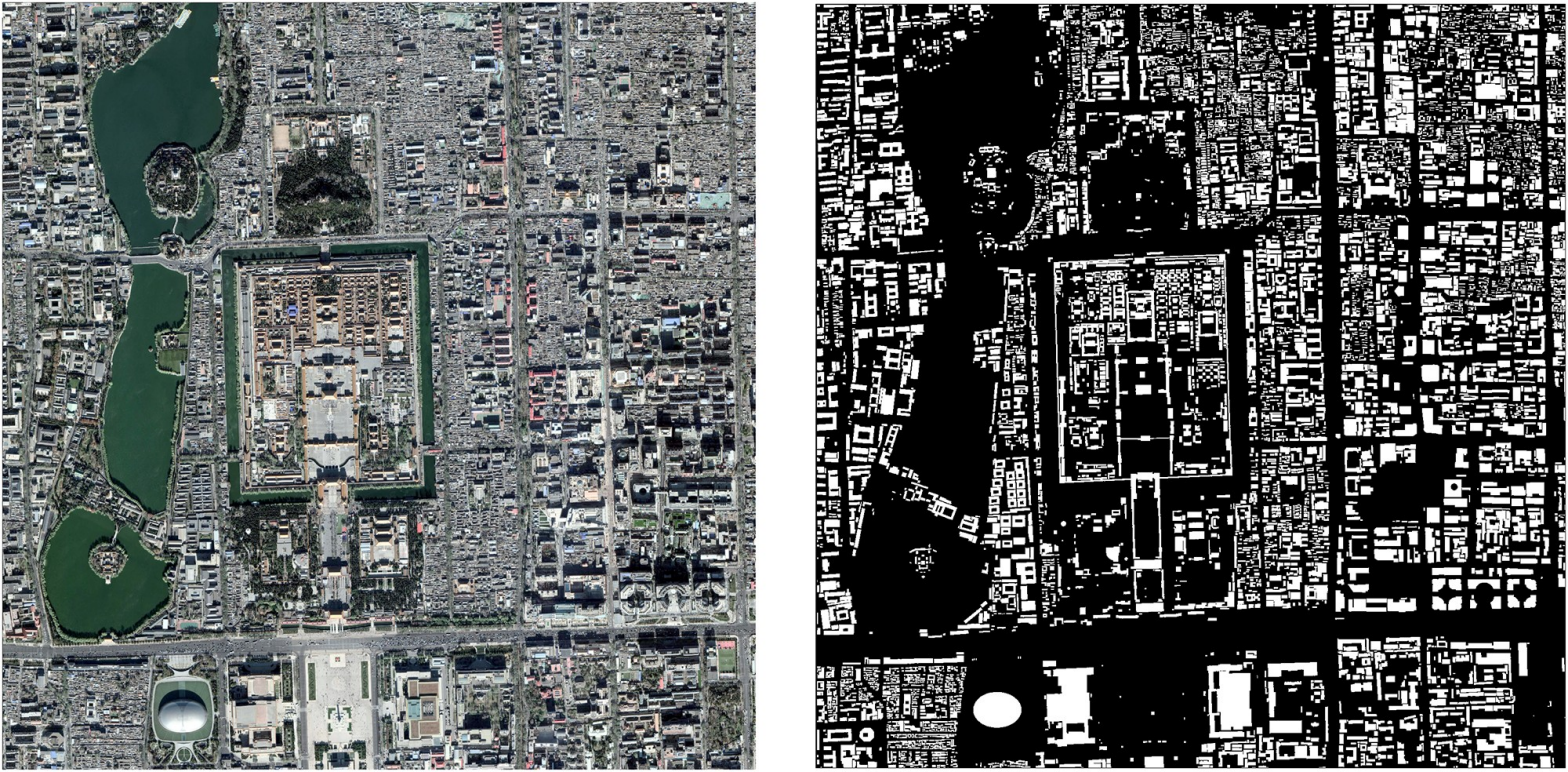
Data used for validation. (a): TIANDITU Imagery. (b): Binary image of building footprint intercepted by Baidu map: Beijing.

The accurate building footprint data acquired for the validation is obtained from the Baidu map (https://map.baidu.com/), the online web map service provided very high resolution building footprint layer as shown in Fig 3. “TIANDITU Imagery” is a comprehensive geographic information service website provided by the China National Surveying and Mapping Geographic Information Bureau. It is loaded with geographic information data covering the whole world in three modes: vector, image and three-dimensional. Therefore, this map is used as a reference. In this study, the building footprint layer selected for validation is acquired in 2017 from Baidu map due to the lack of data in 2014.

## Methods

### Data processing

The building footprint layer of Baidu map was download and converted to raster data with the same resolution as the GHSL built-up area products. One problem in the spatial alignment of Baidu map with GHSL data is the various local projections of Baidu maps compared to a consistent global projection system of GHSL. We proposed and implemented a specific procedure to address this issue by the following five steps:

1. Vector data generation: Obtain the building footprint data of Baidu map, get the binary map of building outline based on the characteristics of houses, and then get the vector data of the building footprint by mosaicking, filtering, vectorization, and geo-registration.
2. Grid generation: Converting the building footprint vector data into raster data of which the spatial resolution is consistent with that of the GHSL built-up areas product requires vector grid data with the consistent grid position of the GHSL built-up areas. Therefore, the grid data of the GHSL built-up areas products are used to generate the required grid data with 250m resolution and 1000m resolution.
3. Intersection operation: The grid of vector data obtained from step 2 is intercepted with the building footprint vector data to overlay the attributes of the building footprint vector data into each grid.
4. Built-up areas calculation: Built-up area is usually expressed as the occupied proportion of the building footprint area in a unit area. The formula for built-up area is:
  $$P_{i,j}=S_{i,j}/S,$$ (1)
  where *P*_*i*,*j*_ is the proportion of built-up area in each grid, *S*_*i*,_ is the building footprint area in the (*i*, *j*)th grid, and *S* is the grid area.
5. Rasterization: Convert the building footprint vector data with building density attributes into raster images, and the built-up areas validation data is produced for this study.

The above steps are applied to process building footprint data over 20 cities in China. A square area with size of 18×18km is selected as the study coverage for each city.

### Validation

In this paper, statistical histogram and linear regression are used to verify the results. From the histogram, the distribution of built-up areas is intuitively illustrated, and the gap between the data can be analyzed. Linear regression is applied to quantitatively analyze the dependency relationship. It is expressed as:

$$y=w^{\prime }x+e,$$ (2)

where e is error and obeys the normal distribution with a mean value of 0 [25].

In this paper, linear regression is used to analyze the relationship between the GHSL built-up area and the built-up area of Baidu maps. And the correlation coefficient is calculated as:

$$r(X,Y)=\frac{Cov(X,Y)}{\sqrt{Var[X]Var[Y]}},$$ (3)

where *Cov*(*X*, *Y*) is the covariance of *X* and *Y*, *Var*[*X*] is the variance of *X*, and *Var*[*Y*] is the variance of *Y*.

## Results and discussion

**Fig 4:**
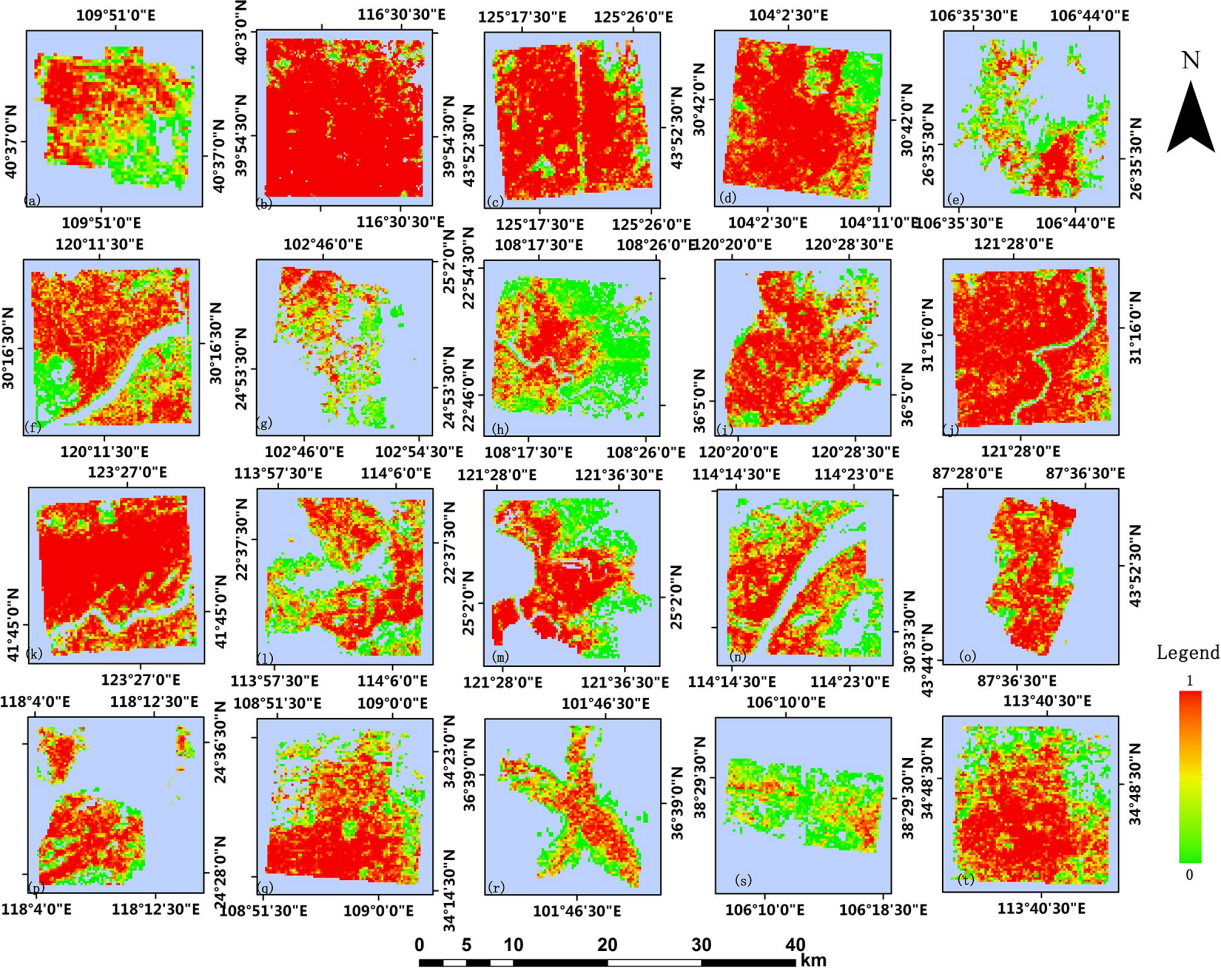
GHSL built-up areas at 250m resolution. Each study area is: (a) Baotou, (b) Beijing, (c) Changchun, (d) Chengdu, (e) Guiyang, (f) Hangzhou, (g) Kunming, (h) Nanning, (i) Qingdao, (j) Shanghai, (k) Shenyang, (l) Shenzhen, (m) Taibei, (n) Wuhan, (o) Urumuqi, (p) Xiamen, (q) Xi’an, (r) Xining, (s) Yinchuan, (t) Zhengzhou.

**Fig 5:**
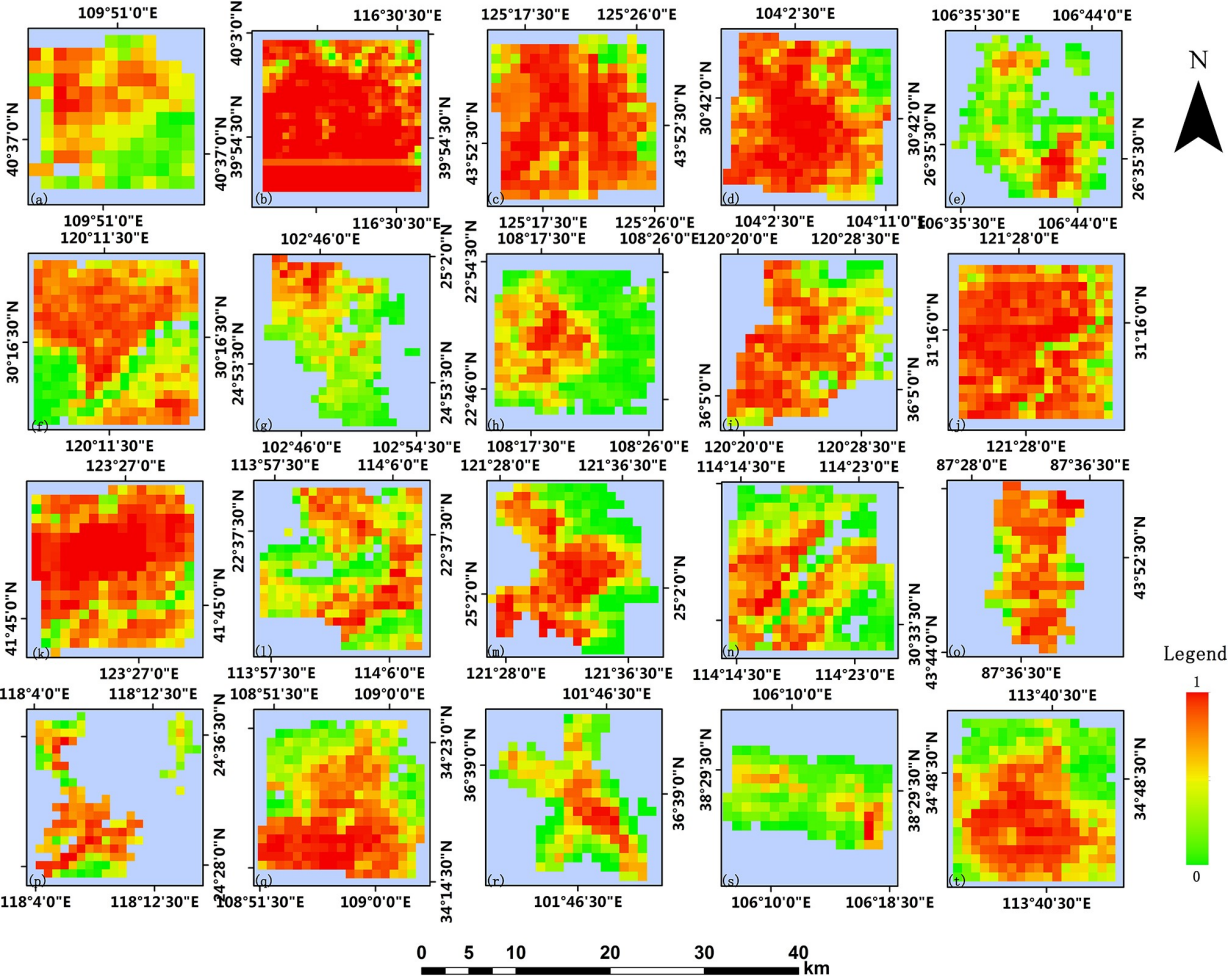
GHSL built-up areas at 1000m resolution.

**Fig 6:**
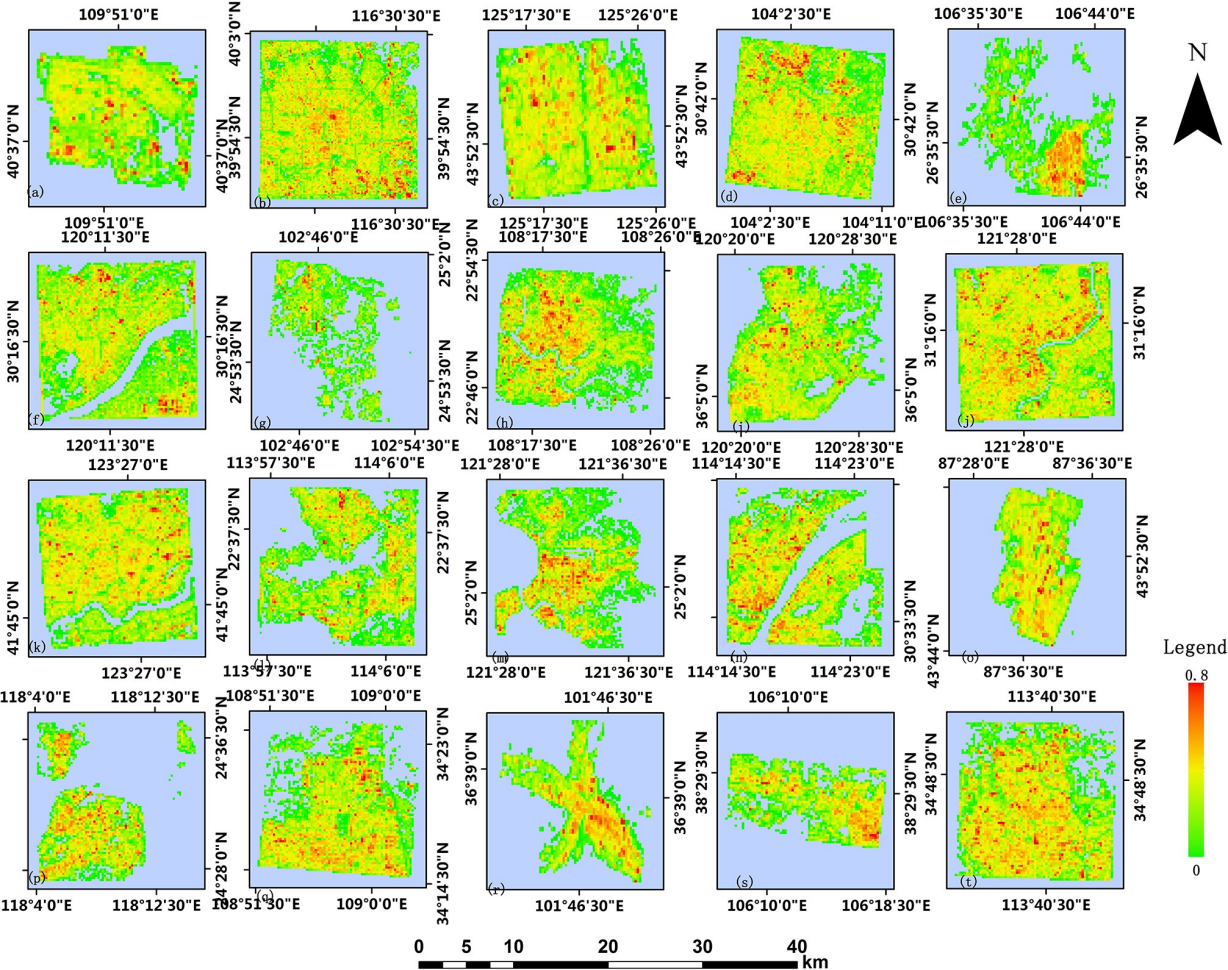
Built-up areas product from Baidu map processed at 250m resolution.

**Fig 7:**
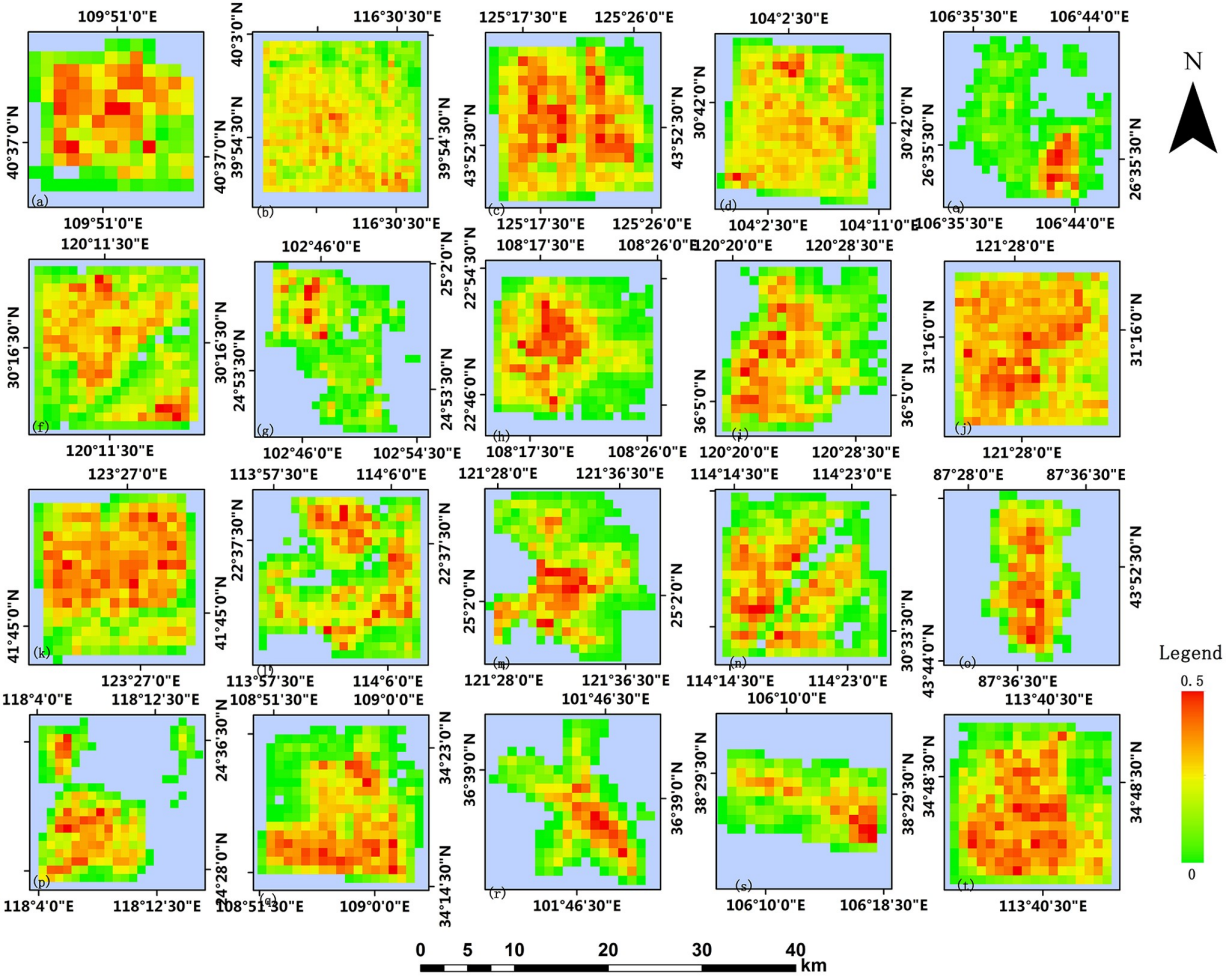
Built-up areas product from Baidu map processed at 1000m resolution.

Based on the 2014 GHSL built-up area products, this paper processes the 2017 building footprint data obtained from Baidu map and obtains built-up areas validation data of 250m resolution and 1000m resolution. Figs 4 and 5 show the GHSL built-up area maps at the 250m resolution and 1000m resolution of 20 study regions in China, respectively. It can be observed that the density of buildings in the urban center is generally higher than that in the suburban and rural areas, which is more apparent in the product with 250m resolution [26]. Figs 6 and 7 show the built-up area maps obtained from Baidu map building outlines at 250m resolution and 1000m resolution in 20 study areas in China, respectively. Similar to the result observed from Figs 4 and 5, the building density in the urban center is higher. Due to the acceleration of urbanization, the city’s building density can vary in different periods. The building density in most cities in China will relatively increase in pace with the development. Generally, the urban suburbs could become the urban core area and the suburbs would expand outwards. Density can change more greatly at the edge of the study area, while in the urban center area it will not change significantly due to limited construction land. However, by comparing Figs 4 and 6, Figs 5 and 7, what we can observe is that the intensities of pixels in GHSL products in 2014 are relatively higher than the values of corresponding pixels in the validation data in 2017 which do not meet the urban development trend. Therefore, it is particularly important to verify the accuracy of GHSL built-up areas data.

**Fig 8:**
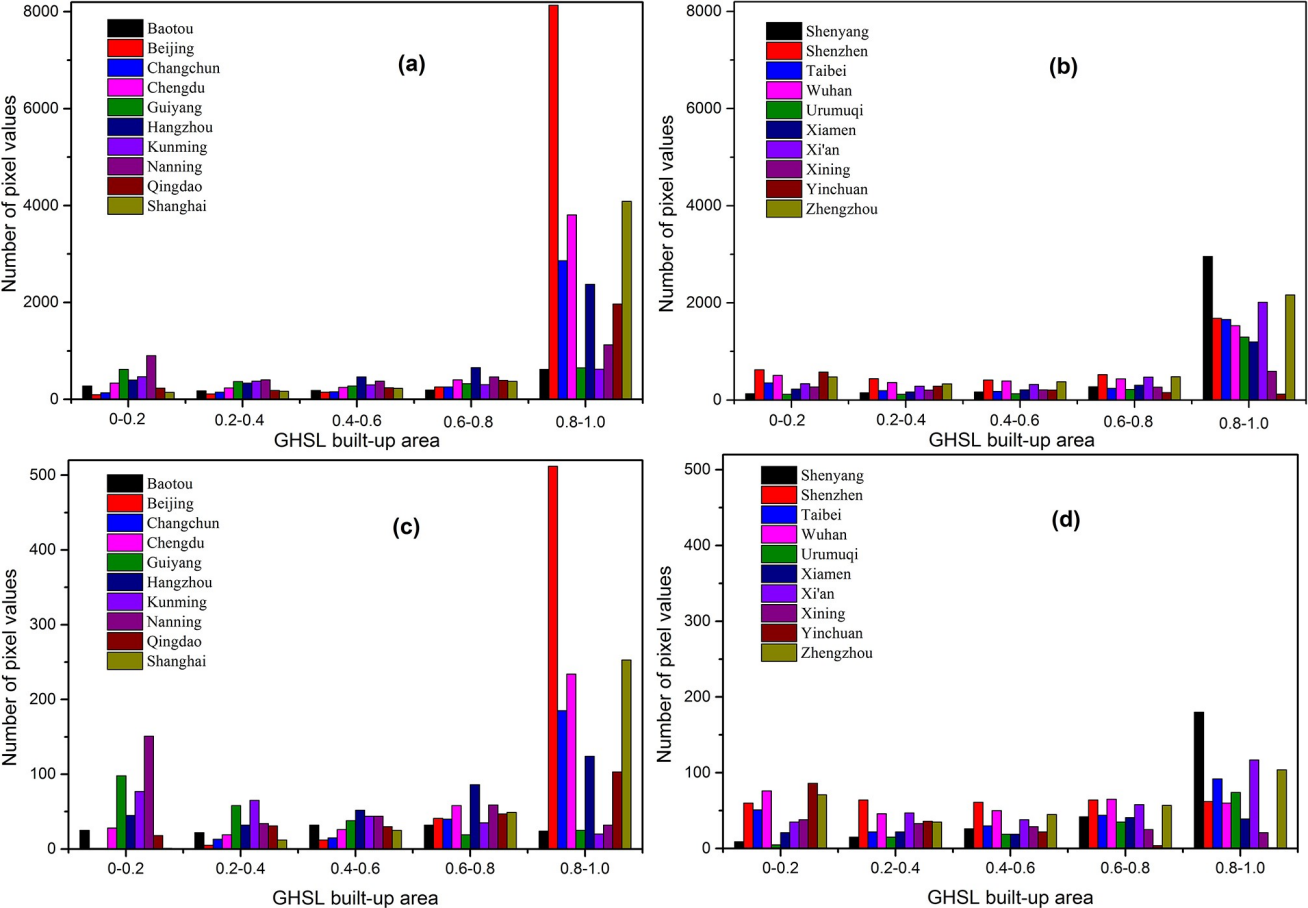
Statistics of GHSL built-up areas pixel values. (a), (b) with 250m resolution; (c), (d) with 1000m resolution.

**Fig 9:**
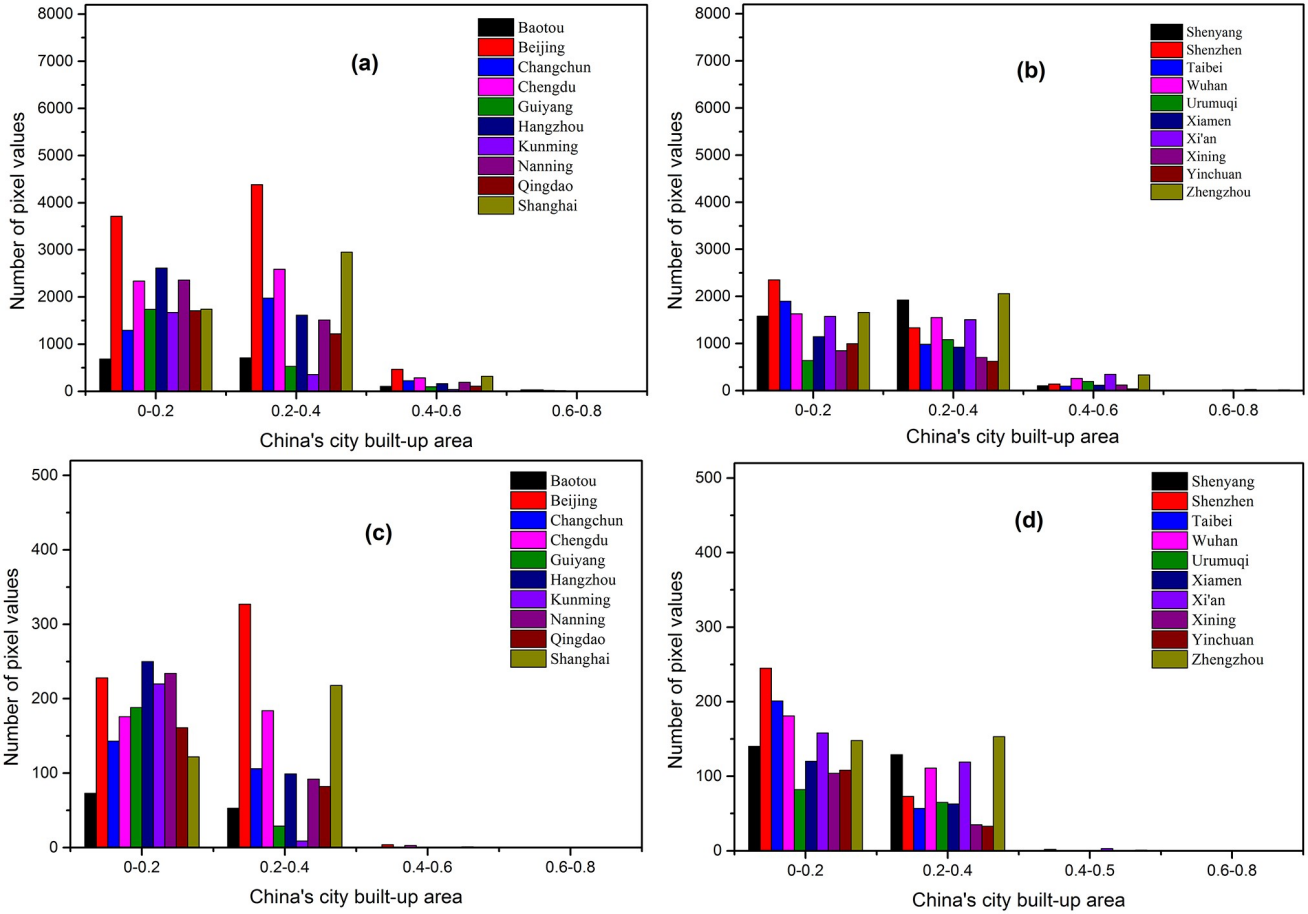
Statistics of pixel values of built-up areas product obtained from Baidu map processing. (a), (b) with 250m resolution; (c), (d) with 1000m resolution.

To reflect the differences between built-up areas of different cities and the differences between GHSL built-up area products (noted as GHSL) and built-up areas of the validation data (acquired from Baidu map and noted as BD) more intuitively. The histograms of different cities at different resolutions are retrieved by performing inter-partition statistics on each pair of GHSL and BD images, as shown in Figs 8 and 9.

Under the same resolution, the bias is existed in the estimations of urban density between BD and GHSL products across different cities. On the one hand, the GHSL built-up area products has a larger number of pixels with an urban building density higher than 0.8 (Fig 8). And the results are similar at GHSL products with different resolutions. It means that the GHSL tends to overestimate large cities (high-density areas) as a whole and underestimate small cities (low-density areas) compared to BD (Figs 8 and 9). The reason is that the GHSL products with 250m and 1000m resolution are interpolated based on the product of 38m resolution which is affected by the mixed pixel impact in the generation of product. Therefore, the building density of regions with low-density buildings will be underestimated and the areas with high-density buildings will be overestimated.

On the other hand, the comparison results of GHSL and BD are different between big cities and small cities under the same resolution. Large cities is generally estimated by GHSL as a high-density area, while small cities with relatively backward economy and population are estimated to be relatively uniform at different building density levels (Fig 8). However, BD generally recognizes the building density of big cities and small cities between 0–0.5, and the building density level of big cities is generally higher than that of small cities in different histogram intervals (Fig 9). This is because the number of pixels of high-density land and low-density land in large cities with higher development level is correspondingly more than that in small cities. Meanwhile, the BD employed for validation (Fig 9) shows that the regions with high building density (above 0.6) correspond to a small number of pixels. Due to the need for part of the land for lighting, urban greening and transportation between buildings, a certain proportion of land will be occupied, thus the histogram distribution of validation data, i.e. BD, is more in line with that of the urban built-up areas in China.

In terms of the comparison between GHSL and BD at different resolutions, both GHSL and BD achieve smoothing effect with the decrease of resolution, reducing the contrast between high-density areas and low-density areas (Figs 8 and 9). For GHSL, the extreme trend of both ends of 250m product is more obvious, while 38m high value data will be smoothed out on 1000m scale due to the change of interpolation unit range, thus reducing the proportion of high value overestimation (Fig 8). The smoothing effect is also applicable to BD products for its data is obtained by converting vector data to grid, thus the high-density area greater than 0.6 is smoothed out with the decrease of resolution to 1000m, and the overall pixel value is concentrated between 0–0.5 (Fig 9). In a conclusion, (1) BD data is more in line with China’s reality in terms of histogram distribution and comparison with the number of pixels across different cities. (2) There is a certain overestimation of the building density of GHSL products in large cities, compared with the estimated density of medium-sized cities, which has a higher consistency with BD. Therefore, BD products are of great value and significance in correcting the overestimation bias of GHSL to big cities.

**Fig 10:**
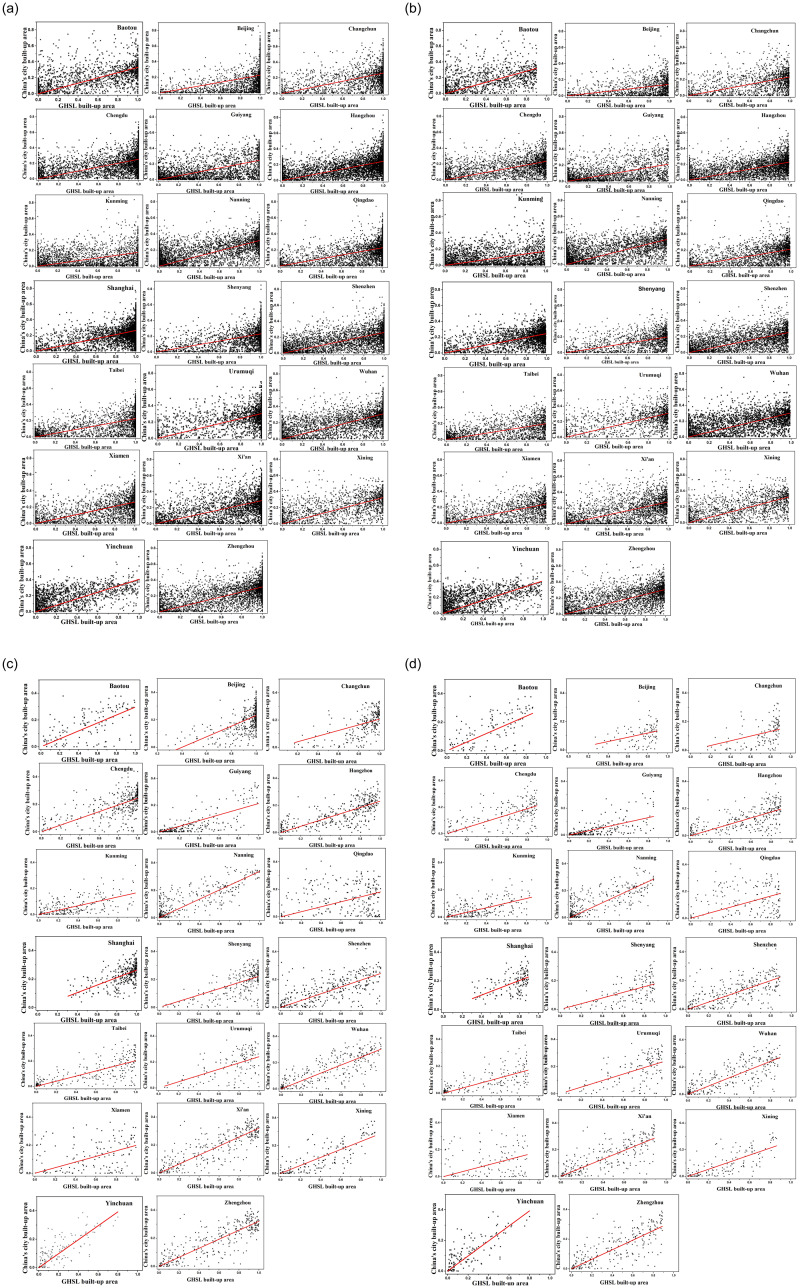
Comparison of GHSL built-up area (X-axis) with processed Baidu map built-up area (Y-axis) in 20 cities. (a) Regression of products with 250m resolution; (b) Regression of products with 250m resolution and saturation values removed; (c) Regression of products with 1000m resolution; (d) Regression of products with 1000m resolution and saturation values removed.

**Fig 11:**
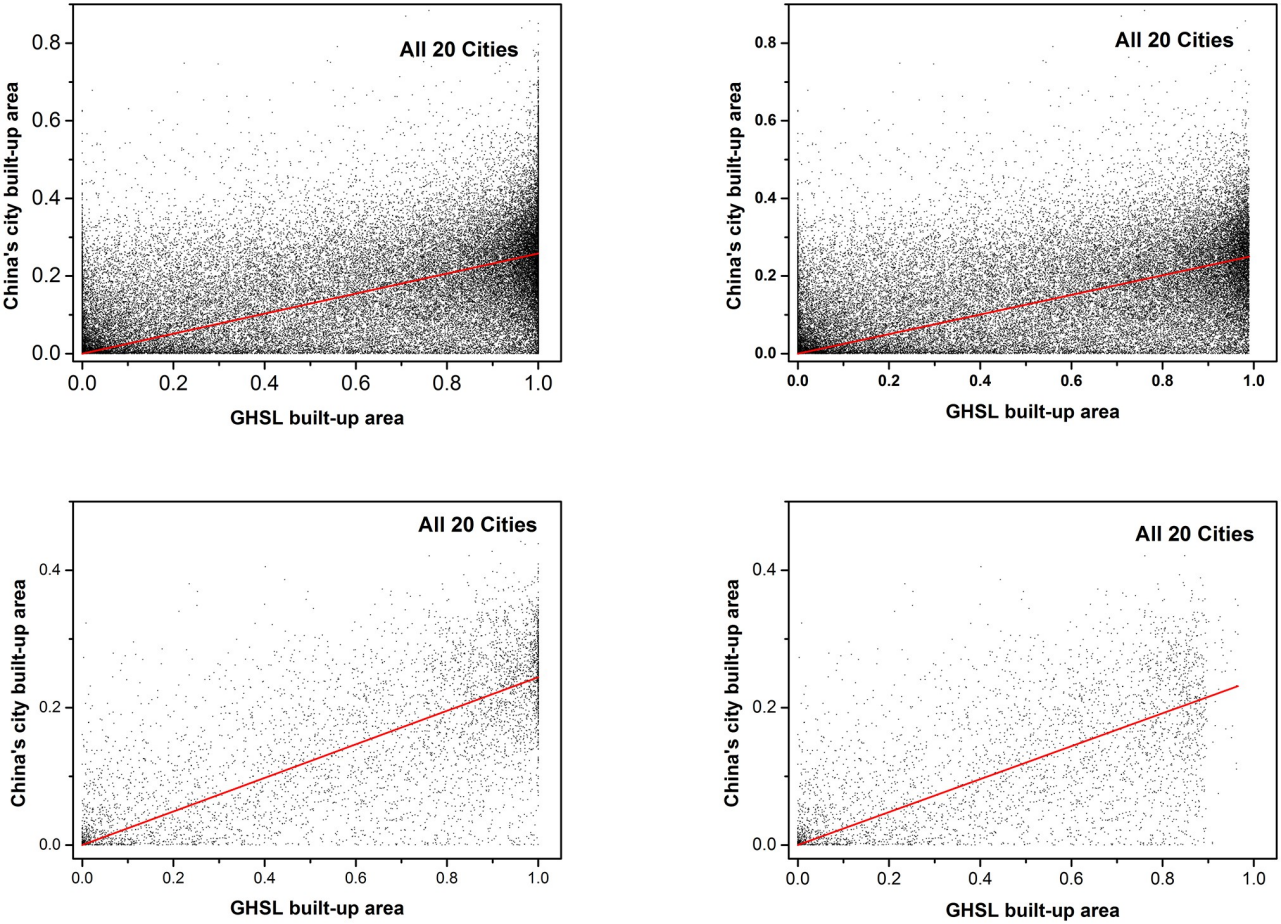
Comparison of GHSL built-up area (X-axis) with processed Baidu map built-up area (Y-axis) in all 20 cities. (a) Regression of products with 250m resolution; (b) Regression of products with 250m resolution and saturation values removed; (c) Regression of products with 1000m resolution; (d) Regression of products with 1000m resolution and saturation values removed.

The pixel values of GHSL built-up area products are higher at different resolutions (250m and 1000m), the data with a saturation value above 0.99 with the resolution of 250m and the data with a saturation value above 0.9 with the resolution of 1000m in GHSL products are considered for quality control according to the distribution of the statistics of GHSL products in all 20 cities. Then the value of GHSL built-up areas and validation data from the cell by pixel with the saturation value removed and the saturation value not removed is compared, as shown in Fig 10 and Fig 11, and the slopes and *R*^2^ are shown in Tables 2 and 3.

**Table 2:** The saturation and unsaturated values of GHSL built-up areas are compared with that of the validating data by the pixel to get the slope and *R*^2^ of 20 cities.

City	250m (saturation)		250m (unsaturated values)		1000m (saturation)		1000m (unsaturated values)	
	Slope1	R_1_^2^	Slope2	R_2_^2^	Slope3	R_3_^2^	Slope4	R_4_^2^
Baotou	0.33	0.73	0.35	0.59	0.30	0.76	0.30	0.73
Beijing	0.23	0.74	0.14	0.55	0.22	0.90	0.15	0.75
Changchun	0.26	0.83	0.23	0.67	0.21	0.85	0.16	0.72
Chengdu	0.25	0.81	0.24	0.70	0.24	0.87	0.23	0.79
Guiyang	0.26	0.63	0.21	0.51	0.21	0.71	0.16	0.59
Hangzhou	0.24	0.77	0.23	0.72	0.23	0.85	0.22	0.80
Kunming	0.17	0.45	0.17	0.41	0.17	0.62	0.17	0.63
Nanning	0.32	0.72	0.32	0.65	0.34	0.83	0.33	0.78
Qingdao	0.23	0.77	0.21	0.70	0.18	0.57	0.21	0.57
Shanghai	0.26	0.86	0.25	0.81	0.26	0.94	0.25	0.90
Shenyang	0.23	0.83	0.20	0.71	0.22	0.86	0.20	0.81
Shenzhen	0.26	0.76	0.25	0.70	0.24	0.79	0.25	0.77
Taibei	0.23	0.76	0.20	0.65	0.20	0.79	0.19	0.74
Wuhan	0.31	0.78	0.30	0.72	0.30	0.86	0.30	0.83
Urumqi	0.30	0.79	0.31	0.76	0.24	0.81	0.24	0.81
Xiamen	0.26	0.77	0.24	0.69	0.20	0.58	0.19	0.47
Xi’an	0.29	0.77	0.28	0.69	0.32	0.85	0.32	0.84
Xining	0.32	0.73	0.33	0.70	0.27	0.83	0.27	0.78
Yinchuan	0.41	0.61	0.41	0.61	0.49	0.80	0.49	0.80
Zhengzhou	0.31	0.82	0.31	0.75	0.32	0.90	0.32	0.84

**Table 3:** The saturation and unsaturated values of GHSL built-up areas are compared with that of the validating data by the pixel to get the slope and *R*^2^ in all cities.

**All cities**	**250m (saturation)**		**250m (unsaturated values)**		**1000m (saturation)**		**1000m (unsaturated values)**	
	**Slope1**	**R**_**1**_^**2**^	**Slope2**	**R**_**2**_^**2**^	**Slope3**	**R**_**3**_^**2**^	**Slope4**	**R**_**4**_^**2**^
	0.26	0.76	0.25	0.66	0.24	0.82	0.24	0.73

**Fig 12:**
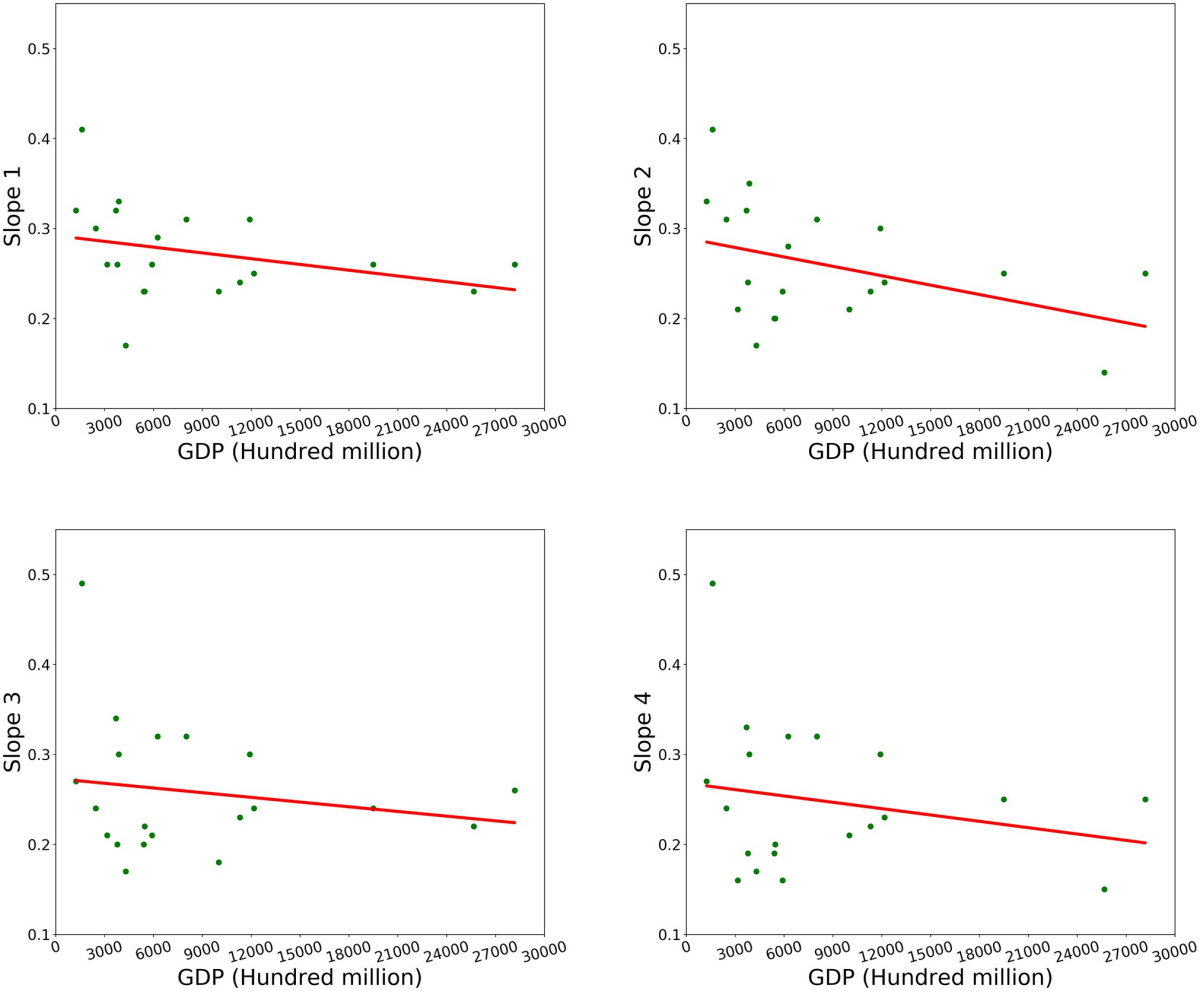
The relationship between the GDP of each city and corresponding regression slopes.

The results of the regression analysis (Figs 10 and 11) suggest that there is no significant difference in the regression slope between the saturated value and the unsaturated value at 250m resolution and 1000m resolution. However, the *R*^2^ of data with saturation values are higher than the *R*^2^ of data without saturation values, and the correlation of data set with 1000m resolution is higher than that with 250m resolution. The comparison between the results of 1000m resolution and 250m resolution products shows that the slope of 1000m resolution product is relatively smaller. In the regression analysis of this study, p < 0.001. From Table 2, we can find the regression parameters of cities with better economic development are lower, such as Beijing, Shanghai, Shenzhen and etc., which may result from the better development of economics, the rapid expansion of construction, the opposite economic level of the city and that the population is not floating in a small number of cities and the speed of construction expansion is relatively slow. Moreover, the trend line between GDP of each city and corresponding regression slope illustrates that the regression slopes of cities with higher GDP are lower, as shown in Fig 12.

In GHSL, the built-up area class is defined as the union of all the spatial units collected by the specific sensor and containing a building or part of it [10]. For the saturation values in the results, it can be explained by the confusion between bare soils in agricultural fields and built-up areas, the appearance similarity between the ridgeline and the building footprints, and that highway (especially asphalt concrete roads) can be misclassified as built-up areas when classifying built-up areas. GHSL built-up area products tend to overestimate the building density over areas with high density since the Landsat image used is of 30m resolution, under which condition single buildings and small settlement patterns surrounded by vegetation may be difficult to identify. The use of the data with high resolution may help in alleviating the problem of confusion between built-up areas and other types of land cover such as artificial open spaces, river gravel and sand dunes [10]. Therefore, the product at 1000m resolution has the best validation result. The validating data used in this paper contains only the building footprints, with no other non-building data existing. In summary, although the *R*^2^ of regression results are not very good, GHSL products have a certain effect on China’s instructions for built-up areas. In terms of the applications of this product in China, it may not be able to meet the high accuracy requirements of building density, but it is suitable for the wide-range study of low resolution.

## Conclusions

Aims to assess the accuracy of GHSL built-up products in China, 20 typical cities across entire China were selected as study sites. The quantitative assessment of the GHSL built-up products was carried out over different types of cities, it is expected to provide a reference of applications of the GHSL products in the dense urban area over East Asia, especially in China. With the report of the assessment, we can conclude that the GHSL built-up products are a promising product for characterizing building density, and the pixel values have a good correlation with the ground truth. However, the comparison with the assessment in the United States and European countries suggest that the significant difference between the regression slopes. Built-uparea per tiles from European and United States as reference data and the GHSL layer has been compared with a regression slope of 0.2164 [17], which is lower than in China (Table 3). The reason for this phenomenon is that the cities in China have much higher building density and height, the shadow effect would be much significant, which will affect the estimation of building density with remotely sensed images [27]. In addition, the Landsat images with 30m spatial resolution were used by JRC in generating the GHSL built-up product. It means individual building and building clusters surrounded by dense vegetation cannot be accurately detected, and the building density was underestimated over where the areas with low buildings. The existence of the mixture pixels which contain signals of roads, bare grounds and buildings would also affect the accuracy of estimation.

In summary, the quantitative assessment over 20 cities in China suggests that the GHSL built-up products have a good correlation with ground truth, however, it is also observed that the products need to be further validated and improved in dense urban areas, especially in East Asia like China. The future work would be focused upon the investigation of estimation models of building density in both sparse and dense urban environment with time series and multi-source data, and eventually develop more generative models for generating building density products over large areas with an operational manner.
